# China NMPA perspective on clinical evaluation of SARS-CoV-2 antibody test reagents in the process of emergency approval

**DOI:** 10.4155/bio-2020-0188

**Published:** 2020-12-16

**Authors:** Yunfeng Lv, Jingyun He, Rongzhi Liu, Yu Gao, Chao Xu, Peng Hu, Shengwei Zheng, Li Fang, Ran Li, Congyin Han, Juanjuan An, Jinchun Dong

**Affiliations:** ^1^Center for Medical Device Evaluation, NMPA, Beijing 100081, PR China

**Keywords:** antibody test reagents, clinical evaluation, COVID-19, emergency approval, SARS-CoV-2, supplementary detection

## Abstract

Coronavirus disease 2019 (COVID-19) pandemic caused by severe acute respiratory syndrome coronavirus 2 (SARS-CoV-2). The SARS-CoV-2 antibody testing an important supplement to nucleic acid testing. In the process of emergency approval, the Center for Medical Device Evaluation of the China National Medical Products Administration released The Key Points of Technical Review for the Registration of SARS-CoV-2 Antigen/Antibody Detection Reagents. The Clinical Study Requirement section of the Key Point has put forward requirements in terms of reference methods and subject enrolment among others, which can ensure that the test results can meet the clinical needs. This article draws on the experience of the China NMPA in evaluating diagnostic reagents used to supplement the gold standard test method in the early stage of an epidemic of an infectious disease, as well as to serve as reference for clinicians and regulators.

The 2019 novel coronavirus has spread rapidly throughout the world. As of 8 July 2020, 11,500,302 [1] people have been diagnosed with infections worldwide. In China, 83,572 cases have been confirmed and 4634 deaths reported [2].

*In vitro* diagnostic reagents are a tool for identifying viral infections as part of the coronavirus disease 2019 (COVID-19) control measures in the various countries, enabling rapid detection and identification of those infected. Nucleic acid testing is a reference method for viral infections. Even though research into nucleic acid testing has revealed that it has a high sensitivity during the first 2 weeks of infection, some patients still show negative results due to sample quality, viral load and reasons during the test. Serological testing will help such patients confirm their disease status and then carry out disease treatment and disease epidemic control accordingly. After 2 weeks, nucleic acid tests show gradually fewer positives as the antiviral therapy takes effect and antibody levels gradually reach the peak detectable level, although the vast majority of infected people can still test positive for IgM and/or IgG. When IgM antibodies turn negative with IgG antibodies persistently positive, this is an indicator of past infections that is effective in the later stages of the disease. These characteristics of serological tests make antibody testing an important supplement to nucleic acid testing [3].

However, the development and verification of antibody detection reagents as an immunological method is more complex than nucleic acid detection reagents. There are huge differences in the recombinant antigens used in different products. Some products make use of recombinant N protein, while other products also employ recombinant S protein. There is a dearth of reference material for the development of reagents due to the lack of research into the human immune response to the novel coronavirus, making it difficult to achieve adequate verification of clinical performance in the laboratory validation process. This situation was particularly prominent in the early stages of the epidemic.

The intended use of the reagent should be adapted to the epidemiological characteristics of the disease. In order to understand the significance of antibody detection reagents in the early stages of an epidemic, their performance should be fully and effectively observed through large-sample clinical studies that can be carried out quickly to evaluate the clinical significance.

At the beginning of the epidemic, some *in vitro* diagnostic reagent manufacturers started research and development of antibody reagents and the Center for Medical Device Evaluation of the China National Medical Products Administration released The Key Points of Technical Review for the Registration of Severe Acute Respiratory Syndrome Coronavirus 2 (SARS-CoV-2) Antigen/Antibody Detection Reagents (hereinafter referred to as ‘Key Points’). Key Points was released with the aim of guiding applicants in conducting laboratory and clinical research, preparing registration application documents and providing reference for technical review departments based on the complementary role of antibody testing with regard to the nucleic acid gold standard testing and the characteristics of the epidemic in the early stage of outbreak. This article will elaborate on the China National Medical Products Administration’s ideas for clinical testing of antibody reagents based on the requirements for clinical evaluation in the Key Points, to provide reference for relevant workers.

## Key points

### Determination of clinical reference methods

SARS-CoV-2 antibody detection reagents are a supplement to nucleic acid detection methods used for auxiliary diagnosis of COVID-19. Therefore, the main evaluation index of clinical trials should be consistency between antibody test results and clinical diagnosis. During the course of clinical testing, the clinical diagnostic criteria should be the basis of the reference system in the diagnosis and treatment plan (guidelines) for the novel coronavirus. In the early stages of an outbreak, there is a lack of internationally accepted diagnostic and treatment programs. Consequently, the Key Points stipulate the Diagnosis and Treatment Guidance issued by the National Health Commission of China as the reference system, including definitions of suspected cases and clinical diagnostic criteria. Criteria for diagnosis include computed tomography (CT) imaging, epidemiological history, clinical symptoms and the results of nucleic acid testing.

### Subject requirements

The enrolled subjects of the clinical trial should be the intended population for the reagent. The subjects to be enrolled in clinical trials of antibody detection reagents for the novel coronavirus should be ‘suspected cases’ of novel coronavirus infection. The criteria for determining suspected cases should be done in reference to the current Diagnosis and Treatment Guide, which includes epidemiological history and clinical manifestations [4]. In addition, suspected cases are identified as either ‘confirmed cases’ or ‘excluded cases’ based on the clinical diagnostic criteria in the ‘Diagnosis and Treatment Guidance’.

Confirmed cases enrolled should include subjects at different stages of the disease, including subjects in the early, middle and late stages of treatment and convalescence.

In addition, a portion of the enrolled subjects should provide continuous samples and serum antibodies in the collected samples should be observed during the entire process from negative to positive.

### Sample size

It is recommended that the size of the enrolled sample be estimated using an appropriate statistical model taking into account the preset values of clinical evaluation indicators such as reagent sensitivity and specificity, as well as related statistical parameters. The presets for the clinical evaluation indicators should be verified based on laboratory research and clinical requirements. Based on the above principles used to determine sample size, it is estimated that the number of confirmed cases should be at least 200 and the number of excluded cases should be at least 300.

There should also be a certain number of enrolled cases of different clinical types and at different stages of the disease course, as well as other cases of respiratory infections, in order to provide a more scientific and comprehensive evaluation of the clinical performance of the product. The sample sizes for each subgroup should meet the statistical requirements.

### Data integrity requirements

Applicants should submit clinical trial data including detailed and complete clinical background of the subject, including the time of onset of clinical symptoms, time of visit, time when samples were taken, sample type, time of the nucleic acid test as well as results, all of which is very important information. In all confirmed subjects, the course of the disease is differentiated based on the time interval from onset to sampling and the early, medium and post treatment recovery periods corresponding to the sampling interval of 1 week, 2 weeks and more for the onset of the disease. Time intervals for samples are an important factor for stratified analysis of the enrolled subjects.

### Statistical analysis

The purpose of clinical trials on these products is to verify whether declared product results are consistent with clinical findings and to evaluate whether product performance meets clinical needs. In general, 2 × 2 contingency tables in diagnostic tests are used to display the results and calculate clinical sensitivity, clinical specificity and confidence intervals are used for statistical analysis of clinical trial data. At the same time, there should be a comprehensive evaluation of the two biomarkers, IgG and IgM, which should be carried out in addition to separate statistical analysis of their test results.

The levels of IgG and IgM will vary with the stage of the disease over the course of the body’s immune response. This necessitates separate stratified analyses based on the characteristics of the two factors of antibody conversion process and subjects’ age and disease course (time interval between sample collection and onset of clinical symptoms), in addition to analysis of the population as a whole.

Continuous samples collected at different times for pneumonia patients with new coronavirus infection should be compared with the results of nucleic acid tests. Evaluate the reported detection ability and window period for the reagents against new coronavirus infections, especially the detection ability in subjects with multiple negative nucleic acid tests, so as to evaluate the clinical value of antibody detection combined with the results of nucleic acid detection.

All inconsistent results from the clinical trials should be fully analyzed in conjunction with the patient’s epidemiological background, clinical symptoms and disease outcome, etc.

## Key considerations

The relevant requirements for clinical research in the Key Points were formulated when the world did not fully understand SARS-CoV-2 during the early stage of the outbreak of the novel coronavirus epidemic. The objective is to understand the performance of the antibody detection kit itself and its role in virus diagnosis through the collection and analysis of clinical trial data in the absence of relevant clinical manifestations and immune response process research results after human infection. The novel coronavirus antibody detection reagent is only intended as an aid to diagnosis of the novel coronavirus and the following are some of our primary considerations in the process of reviewing such an intended use.

### Why do the clinical trials need to be conducted at three centers?

Clinical trials of novel coronavirus antibody detection reagents should be conducted in at least three clinical trial facilities. When there is a disease outbreak, there is no solid foundation for research into differences between virus types, different patients and regions, such as differences in genotype, conclusions based on observations, whether the virus has mutated, etc. For such inclusive studies of reagent performance, we believe that a clinical trial split between at least three clinical trial facilities can provide preliminary confirmation of such aspects of performance.

### Why choose suspected cases?

Suspected cases are the intended target for novel coronavirus antibody detection reagents, so the criteria for determining suspected cases should be used as the enrollment criteria for the clinical trials. The criteria for suspected cases of novel coronavirus are based on both epidemiological history and clinical symptoms in the various editions of the Diagnosis and Treatment Guidance in China. Such clinical symptoms include fever, respiratory symptoms, findings from pneumonia imaging, as well as abnormal leukocyte or lymphocyte counts. A positive nucleic acid test result means a suspected case is a confirmed case. A suspected case can be judged as an excluded case when the temperature of the suspected case returns to normal for more than 3 days, respiratory symptoms are significantly improved, the acute CT exudative chest lesions are significantly improved and viral nucleic acid tests are negative twice consecutively (with an interval between samples of at least 24 h). It is very important that suspected cases act as the enrollment criteria, as opposed to using the results of subjects who have been confirmed/excluded as the enrollment criteria. If the subject did not have a ‘confirmed’ or ‘excluded’ result when enrolled, the clinical diagnosis of the subject was ‘blind’ upon enrollment. Since the nucleic acid test result is the final determining factor, the ability to resolve ‘suspected cases’, especially for those with negative results in early stage testing or during continuous sampling, is an important indicator of clinical performance in supplementing nucleic acid detection reagents.

### Why is it necessary to enroll patients at different stages of the disease?

IgM antibodies are markers of the acute stage of infection, typically appearing early in the infection and disappearing after some time. IgG antibodies typically appear later than IgM antibodies, but they test positive for a longer period of time and are a sign of past infection. Although infection by different pathogens as well as different reagents may result in differences in detection rates, infection should conform to this basic principle of immune response. Even if product performance for IgM and IgG can satisfy clinical requirements in the general population, when the results of stratified analysis based on the disease progress do not conform to the basic pattern of immune response described above, the applicant still needs to perform further analysis of the product performance in order to identify the cause of the noncompliance.

The lack of a clear understanding of changes in human antibodies during the course of the immune response to novel coronavirus infection makes it difficult to anticipate the standard of clinical performance to be satisfied by the reagent for it to be considered to meet clinical needs. Therefore, studying changes in antibodies in various stages of the disease is an important means of evaluating changes in the clinical performance of a product.

### Why we require the inclusion of continuously monitored cases?

In addition to requiring suspected cases based on a single sample test, we also require cases that are continuously monitored through follow-up. These cases will have provided samples for testing on at least two occasions with the corresponding test results. Continuous monitoring of enrolled subjects allows for greater clarity on the course of changes in antibodies as well as the ability of antibody tests to supplement nucleic acid tests as described earlier.

### Why clinical diagnosis is used as the reference standard?

As a new assay reagent, its performance should be evaluated on the basis of clinical sensitivity and specificity, so the reference system selected should be based on clinical diagnosis, which we call the ‘clinical reference standard’ or the ‘gold standard’. Each product, on the other hand, should have the same reference system, so that clinical performance between products has comparability. As a new product, having clinical diagnosis as the reference criterion allows for a more accurate and objective evaluation of clinical performance in the absence of any mature clinical performance requirements.

### How we perform statistical processing of results?

The two main evaluation indicators for diagnostic tests: clinical sensitivity and clinical specificity. When evaluating the clinical performance of products, we need to focus not only on the sensitivity and specificity in all enrolled samples, but also on clinical performance in the subgroups stratified based on the disease stage. The reagent should show good sensitivity and specificity as well as a good confidence interval when there is no bias in the enrolled cases. In general, sensitivity is at least 80% and specificity at least 95%. At the same time, the sensitivity and specificity to IgM and IgG antibodies in each subgroup based on disease stage should conform to the characteristic level of antibody change over the course of the disease. It is also important to evaluate whether the course of changes in antibodies in continuously monitored cases conforms to the pattern of antibody changes.

## Review practice

Based on the above requirements for products, we have identified a number of products with defective performance during our actual review process, including inadequate sensitivity and specificity. Some companies have redeveloped products, made adjustments to raw materials, reaction systems, production processes, etc. based on clinical trial data in order to make product performance indicators meet requirements.

If sensitivity is not good during analysis of the total population, for example, below 80%, then the distribution of the enrolled subjects should first be analyzed. If the distribution of the subgroups based on disease stage is relatively balanced and in line with the expected distribution among the population, the results at this point reflect defects in product performance. Additional results should be incorporated, as well as laboratory findings when necessary, to analyze the cause of the defect and determine whether the product needs to be optimized.

If the rate of subjects testing positive for the antibody is abnormally high in the early stage of the disease, it may be due to errors in subject information or they could be false positives due to poor specificity of the reagent. If the positive rate of antibodies is low in the middle of the disease, it could be due to insufficient sensitivity of the reagent. At the same time, a premature reduction in the rate testing positive for IgG antibodies in later stages of the disease could also indicate poor reagent sensitivity. There are also corresponding patterns for antibodies in convalescent cases, where IgG is positive and IgM can be positive or negative.

The results of single instances of IgM and IgG tests can be influenced by a number of factors. First, the immune response in human body follows a natural progression after infection with the virus, manifesting in order as IgM-/IgG-, IgM+/IgG-, IgM+/IgG+ and IgM-/IgG+. Therefore, the two antibody tests used together can complement each other to improve the efficiency of clinical diagnosis. If one of the two antibodies tests positive, this indicates the potential for infection. Antibody tests will show different results in different disease stages. The proper use of antibody testing will play a positive and important role in the diagnosis of infection and judging the course of the novel coronavirus [5,6].

Continuous serological monitoring is recommended for suspected cases and those in close contact with cases, even if IgG and IgM initially test negative. Patients testing as IgM+/IgG- should be subject to rigorous monitoring and effective patient isolation measures, along with continuous nucleic acid and antibody testing. When IgM antibodies test as continuously positive and IgG antibodies test positive, the patient should be considered to be a new case of novel coronavirus infection. It should be noted that a single IgM- or IgG-positive test result should not be used independently as a basis for diagnosis of novel coronavirus infection. Dynamic changes in antibody test results, especially in patients in the early stage, will provide more compelling evidence for the diagnosis and progression [7].

In addition, SARS-CoV-2 total antibody test reagents can be clinically evaluated based on the methodology described in this paper.

## Limitations

With regard to detecting viruses, antibody tests complement the nucleic acid gold standard test and antibody test results have different meanings and limitations at different stages of the disease epidemic. The method of clinical evaluation of antibody reagents described in this article is mainly for premarket clinical research on antibody detection reagents intended for clinical diagnostic purposes in the early stage of an outbreak and when SARS-CoV-2 is not fully understood globally. Antibody testing products only for clinical diagnosis purposes cannot be used for assessing subjects’ immune status, neutralization of pathogens, vaccine preparation and even plasma therapy, etc.

In clinical practice, at the start of an outbreak, if a subject tests positive for antibodies even if nucleic acid test results are negative, the subject should be given attention, immediately isolated and subject to further tests, thereby further demonstrating the important diagnostic value of antibody reagents. However, in the course of clinical practice, the results of antibody tests alone cannot be used in isolation for diagnosis of novel coronavirus infections due to the limitations of antibody tests. At the same time, because the mechanism of serology (antibody) in resistance to pathogens is not yet fully understood, the results of the tests are not sufficiently representative of infection status.

On the basis of the requirements outlined in the ‘The Key Points’, the China National Medical Products Administration approved the first new coronavirus antibody detection reagent for sale on the market on 22 February. As of 20 May, a total of 18 antibody detection reagents have been approved via the emergency approval process. The approved products make use of colloidal gold immunochromatography technology and chemiluminescent technology. Based on the sensitivity and specificity as determined by clinical trial, we still conservatively determine the intended use of these products to be as follows:

The kit is intended to be used only as a supplementary indicator for suspected cases that have tested negative for coronavirus nucleic acid or for use in conjunction with nucleic acid testing for the diagnosis of suspected cases and is not intended to be a basis for diagnosis or exclusion of pneumonia in cases of infection with the novel coronavirus and is not intended for screening of the general population. A positive test result requires further confirmation, while a negative test is not sufficient to rule out the possibility of infection. The results of this kit are for clinical reference only and it is recommended that a comprehensive analysis of any disease be performed in conjunction with the patient's clinical manifestation and other laboratory tests. The product is intended for use in healthcare facilities only.

## Conclusion

Nucleic acid testing is a reference method for viral infections. However, some patients still show negative results due to sample quality, viral load and reasons during the test. Antibody testing is an important supplement to nucleic acid testing. The main evaluation index of clinical trials should be consistency between antibody test results and clinical diagnosis. The subjects to be enrolled in clinical trials of antibody detection reagents for the novel coronavirus should be ‘suspected cases’ of novel coronavirus infection. Confirmed cases enrolled should include subjects at different stages of the disease, including subjects in the early, middle and late stages of treatment and convalescence. It is necessary to separate stratified analyses based on subjects’ age and disease course, in addition to analysis of the population as a whole. Because the mechanism of serology (antibody) in resistance to pathogens is not yet fully understood, the results of the tests are not sufficiently representative of infection status. Therefore, the kit is intended to be used only as a supplementary indicator for suspected cases that have tested negative for coronavirus nucleic acid or for use in conjunction with nucleic acid testing for the diagnosis of suspected cases and is not intended to be a basis for diagnosis or exclusion of pneumonia in cases of infection with the novel coronavirus and is not intended for screening of the general population.

## Future perspective

As people’s awareness of SARS-CoV-2 is promoted, antibody detection will extend from the auxiliary detection of nucleic acid in the early stage of epidemic outbreak to more application directions, including epidemiological surveillance, immune status assessment, vaccine preparation and even plasma therapy. Aiming at various application directions, there will be different evaluation methods to demonstrate product utility through different clinical trial designs. In clinical trial design, the principles of epidemiological research and clinical trial design should be followed qualify in aspects of the main evaluation index and the exclusion criteria of the subjects so as to ensure that the results are clearly causal to the clinical trial hypothesis.

The novel coronavirus has ravaged the entire world and represents an unprecedented catastrophe in human history. The first objective, in the face of an epidemic of a highly infectious and highly pathogenic virus, is to control the initial outbreak and eradicate the disease. The methods for clinical evaluation of reagents used, the main evaluation indicators and their clinical significance will be different from products used against the background of a general epidemic. This article draws on the experience of the China National Medical Products Administration in evaluating diagnostic reagents used to supplement the gold standard test method in the early stage of an epidemic of an infectious disease, as well as to serve as reference for clinicians and regulators.

**Executive summary**

The 2019 novel coronavirus has spread rapidly throughout the world. Nucleic acid testing is a reference method for viral infections, but some patients still show negative results due to sample quality, viral load and reasons during the test. Antibody testing is an important supplement to nucleic acid testing. This article will elaborate on the China National Medical Products Administration’s ideas for clinical testing of antibody reagents based on the requirements for clinical evaluation in the The Key Points of Technical Review for the Registration of SARS-CoV-2 Antigen/Antibody Detection Reagents (hereinafter referred to as ‘Key Points’), to provide reference for relevant workers.

**Key points:**

**Determination of clinical reference methods**

- The main evaluation index of clinical trials should be consistency between antibody test results and clinical diagnosis.

**Subject requirements**

- The subjects to be enrolled in clinical trials of antibody detection reagents for the novel coronavirus should be ‘suspected cases’ of novel coronavirus infection. Confirmed cases enrolled should include subjects at different stages of the disease. In addition, a portion of the enrolled subjects should provide continuous samples.

**Sample size**

- The size of the enrolled sample be estimated taking into account the preset values of clinical evaluation indicators such as reagent sensitivity and specificity, as well as related statistical parameters.
- There should also be a certain number of enrolled cases of different clinical types and at different stages of the disease course, as well as other cases of respiratory infections. The sample sizes for each subgroup should meet the statistical requirements.

**Data integrity requirements**

- Applicants should submit clinical trial data including detailed and complete clinical background of the subject, including the time of onset of clinical symptoms, time of visit, time when samples were taken, sample type, time of the nucleic acid test as well as results, all of which is very important information.

**Statistical analysis**

- In general, clinical sensitivity, clinical specificity and confidence intervals are used for statistical analysis of clinical trial data. At the same time, there should be a comprehensive evaluation of the two biomarkers, IgG and IgM, which should be carried out in addition to separate statistical analysis of their test results.
- The levels of IgG and IgM will vary with the stage of the disease over the course of the body’s immune response. This necessitates separate stratified analyses based on the subjects’ age and disease course, in addition to analysis of the population as a whole.

**Key considerations**

- We believe that a clinical trial split between at least three clinical trial facilities can provide preliminary confirmation of such aspects of performance.
- The ability to resolve ‘suspected cases’ with negative results in early stage testing or during continuous sampling, is an important indicator of clinical performance in supplementing nucleic acid detection reagents.
- Continuous monitoring allows for greater clarity on the ability of antibody tests to supplement nucleic acid tests as described earlier.
- In general, sensitivity is at least 80% and specificity at least 95%.

**Review practice**

- Based on the above requirements for products, we have identified a number of products with defective performance during our actual review process, including inadequate sensitivity and specificity.
- When evaluating the clinical performance of products, we need to focus not only on the sensitivity and specificity in all enrolled samples, but also on clinical performance in the subgroups stratified based on the disease stage and age.
- The results of single instances of IgM and IgG tests can be influenced by a number of factors. The two antibody tests used together can complement each other to improve the efficiency of clinical diagnosis. If one of the two antibodies tests positive, this indicates the potential for infection. SARS-CoV-2 total antibody test reagents can be clinically evaluated based on the methodology described in this paper.

**Limitations**

- The method of clinical evaluation of antibody reagents described in this article is mainly for pre-market clinical research on antibody detection reagents intended for clinical diagnostic purposes in the early stage of an outbreak and when SARS-CoV-2 is not fully understood globally. Antibody testing products only for clinical diagnosis purposes cannot be used for assessing subjects' immune status, neutralization of pathogens, vaccine preparation and even plasma therapy, etc.

**Future perspective**

- As people’s awareness of SARS-CoV-2 is promoted, antibody detection will extend to more application directions. In clinical trial design, the principles of epidemiological research and clinical trial design should be followed qualify in aspects of the main evaluation index and the exclusion criteria of the subjects so as to ensure that the results are clearly causal to the clinical trial hypothesis.
- This article draws on the experience of the China National Medical Products Administration in evaluating diagnostic reagents used to supplement the gold standard test method in the early stage of an epidemic of an infectious disease, as well as to serve as reference for clinicians and regulators.

## References

[1] WHO. Coronavirus Disease 2019 (COVID-19) Situation Report 169 (2020). https://www.who.int/docs/default-source/coronaviruse/situation-reports/20200707-covid-19-sitrep-169.pdf?sfvrsn=c6c69c88_2

[2] National Health Commission of the People's Republic of China. Update on Epidemic Situation of COVID-19 by 24:00 on July 7, 2020 (2020). http://www.nhc.gov.cn/xcs/yqfkdt/202007/5ef5e925beff4c1fa5c833edca25cc64.shtml

[3] LI X, LIU G, LI W Application value of chemiluminescence immunoassay in the detection of 2019-nCov antibodies. J. Shandong University (Health Sciences) 58(5), 46–50 (2020).

[4] National Health Commission of the People's Republic of China. Diagnosis and Treatment Scheme of COVID-19 (Version 7) March 4, 2020 (2020). http://www.nhc.gov.cn/yzygj/s7653p/202003/46c9294a7dfe4cef80dc7f5912eb1989.shtml 10.21147/j.issn.1000-9604.2020.04.01 PMC7491549 32965276

[5] Yu F, Du L, Ojcius DM Measures for diagnosing and treating infections by a novel coronavirus responsible for a pneumonia outbreak originating in Wuhan, China. Microbes Infect. 22(2), 74–79 (2020). 32017984 10.1016/j.micinf.2020.01.003 PMC7102556

[6] GUAN M Expert consensus on clinical application of coronavirus nucleic acid and antibody detection. Int. J. Lab. Med. 41(14), 1665–1669 (2020).

[7] Shu H, Wang S, Ruan S Dynamic changes of antibodies to SARS-CoV-2 in COVID-19 patients at early stage of outbreak. Virologica Sinica 2020, 1–8 (2020). 10.1007/s12250-020-00268-5 PMC7383121 32720214

